# Identification and functional analysis of the BIM interactome; new clues on its possible involvement in Epstein–Barr Virus-associated diseases

**DOI:** 10.1186/s40709-015-0037-0

**Published:** 2015-12-23

**Authors:** Erasmia Rouka, Despoina Kyriakou

**Affiliations:** Department of Transfusion Medicine, University Hospital of Larisa, Viopolis, 41110 Larisa, Greece

**Keywords:** BIM interactome, EBV, Functional analysis, Neurodegenerative disorders, Infectious agents

## Abstract

Epigenetic deregulation is a common feature in the pathogenesis of Epstein–Barr Virus (EBV)-related lymphomas and carcinomas. Previous studies have demonstrated a strong association between EBV latency in B-cells and epigenetic silencing of the tumor suppressor gene BIM. This study aimed to the construction and functional analysis of the BIM interactome in order to identify novel host genes that may be targeted by EBV. Fifty-nine unique interactors were found to compose the BIM gene network. Ontological analysis at the pathway level highlighted infectious diseases along with neuropathologies. These results underline the possible interplay between the BIM interactome and EBV-associated disorders.

It is well established that the epigenetic interplay between Epstein–Barr Virus (EBV) and the host contributes to the pathogenesis of EBV-related diseases [1, 2]. Previous studies have revealed that EBV latency in B-cells leads to the epigenetic silencing of the BCL2-Like 11 apoptosis facilitator (BIM) [3, 4]. This study presents a bioinformatic analysis towards the identification and functional prediction of BIM interacting partners so as to indicate new, potential host targets of EBV. The gene network of BIM was constructed by interrogating the following three databases: Biogrid (http://thebiogrid.org), String (http://string-db.org) and GeneMania (http://www.genemania.org). The Biogrid database is a repository of genetic and protein–protein interactions that are curated from the primary biomedical literature for all major model organism species [5]. String is a database of known and predicted protein interactions including both direct (physical), and indirect (functional) associations [6]. GeneMania is a prediction server that generates hypotheses about gene function, analyzes gene lists and prioritizes genes for functional assays [7]. Subsequently, the Human Gene Compendium-GeneCards (http://www.genecards.org) was used in order to assess the description of the retrieved genes. GeneCards is an integrated database of human genes that provides concise genomic related information, on all known and predicted human genes [8]. BIM interactors that were found in all three databases were then entered as a list in the GeneCodis3 database (http://genecodis.cnb.csic.es/) for functional enrichment analysis of Gene Ontology (GO) annotations relative to cellular pathways. GeneCodis is a tool for modular and singular enrichment analysis oriented to integrate information from different sources [9]. Annotations selected were KEGG and PANTHER pathways. The *p* values were obtained through Hypergeometric analysis corrected by the False Discovery Ratio (FDR) method. The option of high confidence/no more than ten interactors was selected in the String database so as to increase the level of certainty.

Fifty-nine unique BIM interactors were identified. Seven of these interactors (MCL1: myeloid cell leukemia 1; BCL2L1: BCL2-like 1; BCL2: B cell CLL/lymphoma 2; DYNLL1: dynein, light chain, LC8-type 1; BCL2L2: BCL2-like 2; MAPK8: mitogen-activated protein kinase 8; BAX: BCL2-associated X protein) appeared in all three databases and were further subjected to functional analysis as the level of confidence for the common genes is much more significant. The modular enrichment analysis identified: (1) pathways related to cancer including apoptosis, (2) pathways related to Amyotrophic Lateral Sclerosis (ALS), and (3) pathways related to other infectious diseases like tuberculosis and toxoplasmosis (Table 1). The singular enrichment analysis of KEGG and PANTHER pathways highlighted: (1) pathways involved in cancer as JAK-STAT and Wnt signalling pathways, (2) pathways related to neurodegenerative disorders [like Huntington’s disease (HD) Parkinson disease (PD), Alzheimer disease (AD) and ALS], (3) pathways involved in infectious diseases like epithelial cell signalling in *Escherichia coli* infection, Chagas disease, Hepatitis C and prion diseases (results not shown).

**Table 1:** Results of the modular enrichment analysis in the GeneCodis3 database

Genes	NGR	TNGR	NG	TNG	Hyp	Hyp*	Annotations
6	107	34,208	6	7	5.67 × 10^−15^	2.84 × 10^−14^	Panther pathways P00006: Apoptosis signalling pathway
3	5	34,208	3	7	5.25 × 10^−11^	8.74 × 10^−11^	KEGG 04141: Protein processing in endoplasmic reticulum KEGG 04722: Neurotrophin signalling pathway KEGG 05200: Pathways in cancer KEGG 05152: Tuberculosis Panther pathways P00006: Apoptosis signalling pathway KEGG 05210: Colorectal cancer
4	37	34,208	4	7	4.04 × 10^−11^	1.01 × 10^−10^	KEGG 05200: Pathways in cancer Panther pathways P00006: Apoptosis signalling pathway
3	9	34,208	3	7	4.40 × 10^−10^	5.51 × 10^−10^	KEGG 05200: Pathways in cancer KEGG 05014: Amyotrophic Lateral Sclerosis (ALS) Panther pathways P00006: Apoptosis signalling pathway KEGG 04210: Apoptosis
3	26	34,208	3	7	1.36 × 10^−08^	1.36 × 10^−08^	KEGG 05200: Pathways in cancer KEGG 05145: Toxoplasmosis Panther pathways P00006: Apoptosis signalling pathway

*p* values have been obtained through Hypergeometric analysis corrected by the False Discovery Ratio (FDR) method. Annotations selected were KEGG and PANTHER pathways

*NGR* number of annotated genes in the reference list, *TNGR* total number of genes in the reference list, *NG* number of annotated genes in the input list, *TNG* total number of genes in the input list, *Hyp* Hypergeometric *p* value, Hyp* corrected hypergeometric *p* value

EBV is a known oncogenic virus associated with 1 % of cancers worldwide [10]. The interactions of EBV with members of the Bcl-2 family like BAX, BCL2, BCL2L1, BCL2L2 and MCL1 in human tumor cells, have been extensively reviewed in Fu et al. [11]. Thus, the prediction that the BIM interactome is involved in cancer pathways was not surprising. The main finding of this study is the indication that several BIM interactors participate in pathways related to neurodegenerative disorders. In such diseases, like AD, PD, ALS and—to a less extent—HD, many studies have highlighted the importance of the immune system and the involvement of neuroinflammation [12]. It has been suggested that the adaptive immune response to EBV represents the key initiating event in PD [13]. Recently, a study has reported on a high level of octapeptide matching between 7 viruses (including EBV), and human brain antigens that, when altered, have been specifically associated with neuropathologies such as ALS, HD and PD [14]. Another important finding arising from the ontological analysis is the prediction that the BIM interactome participates in pathways associated with other infectious agents. The cooperative contribution of HIV, malaria and EBV in lymphomagenesis has long been established while the possibility of synergy between EBV and *Helicobacter pylori* in the pathogenesis of gastric carcinomas has recently begun to unfold [15]. Any possible association of EBV with other infectious diseases revealed by the functional analysis remains to be studied.

Cumulatively, by using contemporary bioinformatic tools this study has demonstrated that the BIM regulatory network consists of 59 unique genes that could be targeted by EBV. Furthermore, it has predicted that several BIM interactors have a central role in pathways associated with neurodegenerative disorders and infectious diseases. The BIM interactome warrants further study, in terms of clinical research, with respect to its interplay with EBV.

## Abbreviations

AD: Alzheimer’s disease
ALS: Amyotrophic Lateral Sclerosis
EBV: Epstein–Barr Virus
HD: Huntington’s disease
PD: Parkinson’s disease
